# Using Single Colors and Color Pairs to Communicate Basic Tastes II: Foreground–Background Color Combinations

**DOI:** 10.1177/2041669516663750

**Published:** 2016-09-20

**Authors:** Andy T. Woods, Fernando Marmolejo-Ramos, Carlos Velasco, Charles Spence

**Affiliations:** Crossmodal Research Laboratory, Oxford University, UK; Gösta Ekman Laboratory, Department of Psychology, Stockholm University, Sweden; Crossmodal Research Laboratory, Oxford University, UK

**Keywords:** color, color pairs, basic tastes, crossmodal correspondences, design

## Abstract

People associate basic tastes (e.g., sweet, sour, bitter, and salty) with specific colors (e.g., pink or red, green or yellow, black or purple, and white or blue). In the present study, we investigated whether a color bordered by another color (either the same or different) would give rise to stronger taste associations relative to a single patch of color. We replicate previous findings, highlighting the existence of a robust crossmodal correspondence between individual colors and basic tastes. On occasion, color pairs were found to communicate taste expectations more consistently than were single color patches. Furthermore, and in contrast to a recent study in which the color pairs were shown side-by-side, participants took no longer to match the color pairs with tastes than the single colors (they had taken twice as long to respond to the color pairs in the previous study). Possible reasons for these results are discussed, and potential applications for the results, and for the testing methodology developed, are outlined.

## Introduction

Research shows that people tend to associate gustatory and nongustatory information in ways that are often surprising (e.g., O’Mahony, 1983). So, for example, people not only match a flavor with a color when they belong to the same object, for example, the aroma or flavor of cucumber with the color green (Garber, Hyatt, & Starr, 2000; Stillman, 1993; Velasco et al., 2015) but also match some more abstract dimensions of sensory information such as basic tastes with color cues (Koch & Koch, 2003; Spence et al., 2015). These matchings, what are often referred to as crossmodal correspondences, refer to the surprising associations that people share between sensory features, attributes, or dimensions of experience in different senses (see Marks, 1978; Parise, 2016; Spence, 2011, 2012, for reviews).. In a recent review of the literature on crossmodal correspondences between colors (or color words) and basic tastes (or taste words), Spence et al. (2015) concluded that, across a number of studies that have been conducted over the past three decades, people consistently associate certain colors with specific basic tastes. Sweet, for example, tends to be associated with colors such as red or pink, sour with yellow or green, salty with white or blue, and bitter with black or other dark colors such as brown or purple (see also Favre & November, 1979; Velasco et al., 2016). One possible reason why such associations arise is that it is actually some specific or general food memory prompted by the color, or indeed even hue, that gives rise to a particular assignment to taste (as suggested in the discussion of Woods, Poliakoff, Lloyd, Dijksterhuis, & Thomas, 2010), rather than the color itself. For example, the color pink may prompt the concept of candyfloss, and it may be this association rather than the color itself that is thought of as sweet (not all of the authors though believe that correspondences are necessarily grounded in specific experiences of environmental objects that possess both features).

Crossmodal matching studies, as well as those studies involving the matching of taste words with colors, suggest that people make reliable associations between tastes and single colors (see Koch & Koch, 2003; O’Mahony, 1983; Spence et al., 2015; Velasco et al., 2016; see also Razumiejczyk, Macbeth, Marmolejo-Ramos, & Noguchi 2015, for recent evidence that taste words can simulate actual gustatory taste experiences). Nonetheless, further research is still needed in order to clarify the extent to which pairs (or triples, etc.) of colors would lead to more consistent taste matchings (e.g., Schifferstein & Howell, 2015), be they either congruent, such as red and pink for sweet, or incongruent, such as red and green for sweet (Woods & Spence, 2016). This empirical question is particularly relevant given that the visual appearance of most foods and drinks does not include only a single color. Instead, they involve various visual characteristics with different ranges of hue, brightness, and saturation (one can even consider how people view a colored food against the background color of the plate; Piqueras-Fiszman, Alcaide, Roura, & Spence, 2012). Therefore, it can be hypothesized that multiple color cues, if chosen appropriately, may provide more information when inferring a specific taste quality.

**Figure 1.**
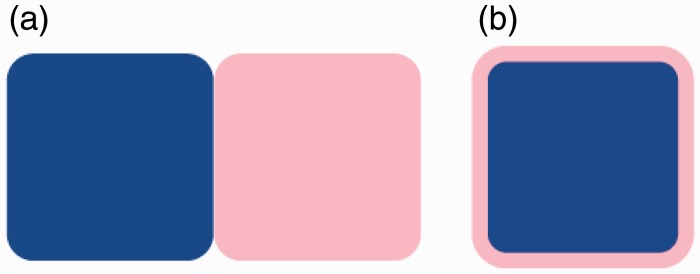
Sample color pairs from Woods and Spence (2016) (a) and as used in the present study (b).

In a recent study, Woods and Spence (2016) presented pairs of colors side-by-side (see Figure 1(a) for an example). The participants had to decide as rapidly as possible which of the four basic tastes (sweet, bitter, salty, or sour) the color pair was best associated with. One of the results to emerge from this study was that some of the 16 color pairs tested better associated with one of the basic tastes than when either of the respective colors was shown individually (e.g., for sourness, the green-yellow color pair). However, on the flip side, the participants took more than twice as long to allocate color pairs, which could be taken to imply some internal reasoning as to which color the participant decided to pair up with a given taste. According to Woods and Spence, it could also mean that the colors are individually assessed in a serial manner and then assimilated, which might well take some time to achieve. A third explanation is that the colors clashed,^1^ which may have interfered with the participants’ rating. The first two explanations imply the involvement, in the study in the Woods and Spence’s article, of a quite different set of (possibly higher order) processes (e.g., operating at a decisional level) than would typically occur in everyday perceptual experience.

Both of the studies reported here are based on the methodology developed in Woods and Spence (2016). In the first experiment though, rather than presenting side-by-side color patches, as in Woods and Spence’s (2016) recent study, we decided to present a foreground–background pair of colors instead (see Figure 1(b) for an example). Our expectation was that such an arrrangement would be somewhat more likely to be treated as a unitary gestalt rather than as two separate elements to be evaluated individually, as may have been the case in Woods and Spence’s study. We tested whether combined color cues versus single colors would result in significant differences in response times when matching the color stimuli with taste words. The prediction was also that the foreground color (in Figure 1(b), blue) would dominate over the background color (pink; henceforth, stimuli are referred to according to their foreground than background color, which here is bluePink) in terms of taste expectations, be it simply because by far the majority of the patch is of the foreground color (more salient), or because, for example, foreground elements tend to be processed sooner than those in the background (Lester, Hecht, & Vecera, 2009). Given this, we predicted that simply reversing the foreground and background colors could well lead to quite different taste expectations. In our second study, we used a different paradigm that we thought would be more sensitive to association differences compared with that used in Experiment 1.

## Experiment 1

### Methods

In both of the studies reported here, we use robust statistics (see Wilcox, 2012 and the R package “WRS”). Specifically, whenever we report means (i.e., averages), we refer to 20% trimmed means (see Wilcox, 2005) and 95% CIs were estimated around means, via the “trimci” function. Percent-bend correlations, here *r_pb_*, and 95% CIs around correlation values were used, which were generated via the “corb” function. One-way robust repeated measures analyses of variance were estimated for comparisons between dependent groups (via the “rmanova” function). The *p* values of post hoc comparisons and multiple pairwise comparisons were adjusted via the Holm-Bonferroni method (Holm, 1979). Categorical data were analyzed via the chi-square test.

### Participants

One hundred participants (62 women; *M*_age_ = 31.3, *SD*_age_ = 11.3, range_age_ = 18–64 years) were recruited from Prolific Academic to take part in the study in return for a payment of 1.00 UK pound. By means of Prolific Academic’s “filter” feature, only those participants who reported having been born in the UK were allowed to take part in the study. The experiment was conducted on November 16, 2015 from 10:50 GMT onwards, over a period of 90 min. The participants completed the study at a rate of 1.13 participant completions per minute (see Woods, Velasco, Levitan, Wan, & Spence, 2015, for a recent methodological overview of Internet-based perception research). The participants took an average of 366 s (95% CI [343, 390]) in order to complete the study. All of the participants in both studies provided their informed consent prior to taking part. The experiments were reviewed and approved by the Central University Research Ethics Committee at Oxford University and were carried out in accordance with the World Medical Association Helsinki Declaration (World Medical Association, 2013).

### Stimuli

Sixty-four color squares (75 × 75 pixels) or circles (diameter 75 pixels), each with a 5-pixel color border, were used as stimuli. The central color and border color consisted of all possible combinations of green, yellow, red, pink, blue, white, black, and purple, the so-called *internet safe* colors (hex codes were 00FF00, FFFF00, FF0000, FFC0CB, 0000FF, FFFFFF, 000000, 800080, respectively). Whether a participant saw square or circular stimuli was determined randomly at the start of each participant’s experimental session. The background against which the stimuli were presented was gray (hex code 7A7A79).

### Apparatus

Given that the experiment was conducted online, the apparatus varied by participant. Nevertheless, the experiment utilized “full screen” mode (i.e., utilizing the entirety of the participant’s monitor), and took place within a 1024 × 768 pixel box in the center of the screen, irrespective of the size of the participant’s monitor. The experiment was conducted on the Internet using the Adobe Flash-based version of Xperiment (http://www.xperiment.mobi).

### Design

A within-participants experimental design was adopted with all of the participants undertaking all 64 of the experimental trials in a random order. There were two dependent variables: The taste word chosen for each color stimulus and the time taken by the participant to decide on that taste word.

### Procedure

**Figure 2.**
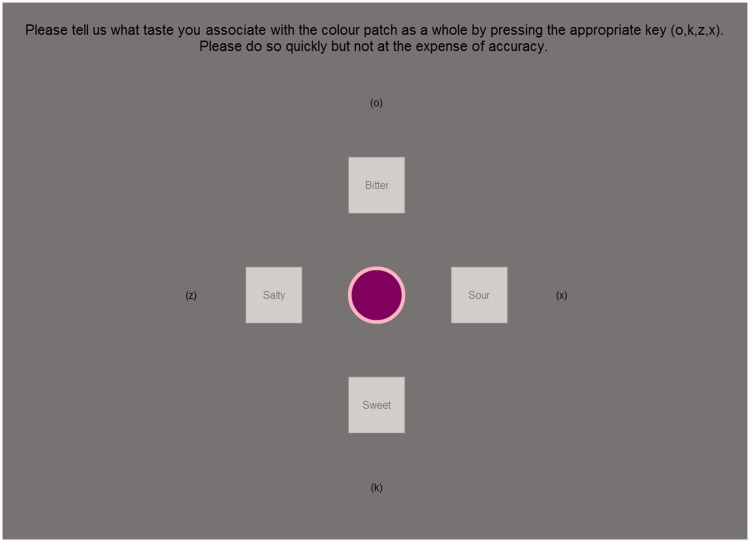
Screenshot illustrating the task used in Experiment 1. The participants had to judge which of the four taste words best matched the color pair shown in the center of the screen by pressing one of four keyboard letters that was closest spatially to the taste they had decided on. The participant could not make a response with the mouse.

The participants had to select the taste word that they felt best represented the color stimulus; the position of the response words, see Figure 2, was randomized across participants. The selection was made by pressing one of four response keys that labeled the responses (o, k, z, or x). The next trial commenced after a 100-ms pause. There were five practice trials before the experiment commenced; the stimuli shown during the practice trials were selected at random from those shown during the actual study. After completing all the trials, the participants were debriefed as to the nature of the study.

### Results and Discussion

**Figure 3.**
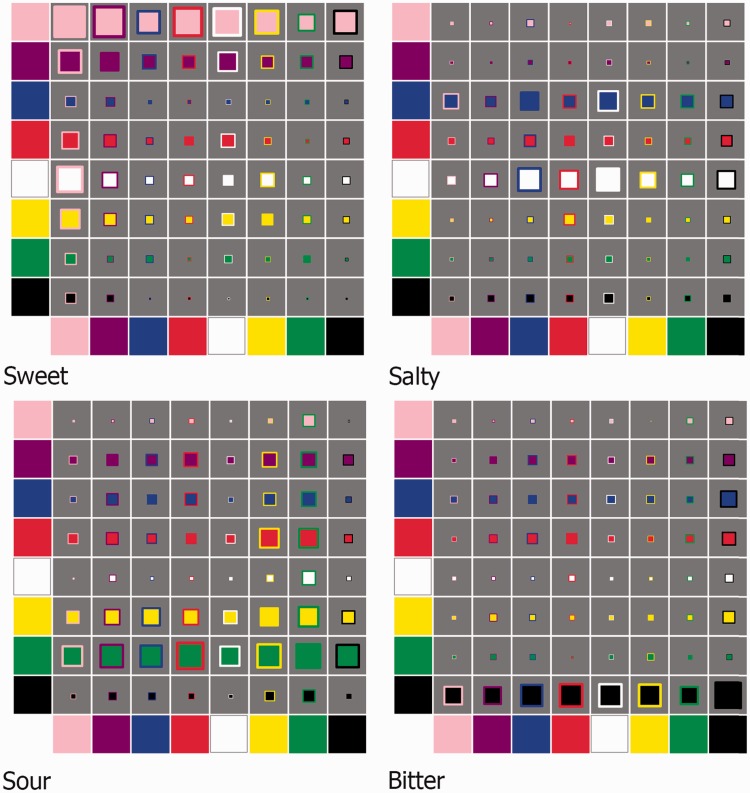
Four color matrix plots depicting the frequency with which the different colors and color pairs were selected for each of the four basic tastes used in the present study. For each plot, the eight colors are shown along the *x*- and *y*-axes. The square color patch within each gray box consists of the color, or colors, that was, or were, shown to participants in order to assign a basic taste to. The size of the color patch, relative to the size of the gray box in which it is presented, represents the number of participants who chose that color patch to reflect the given basic taste (the length of the patch as compared with the length of the gray box representing the ratio of participants choosing that particular taste for the given color). So, for example, if the patch were to fill the entire gray box, this would imply that *every* participant selected the given color for the particular matrix’s taste; if the gray box were to be empty, however, this would imply that *none* of the participants selected that color.

The number of participants selecting each taste for each given color stimulus is shown in Figure 3. Descriptively, certain of the stimuli would appear to be linked by participants with a given taste much more often than the others. So, for example, the single black color patch (or, in other words, black with a black border) would seem to be the stimulus that is most strongly linked with bitterness. Meanwhile, the green stimulus with a red border seems most strongly linked with sourness (68 individuals associated it such); note that the inverse of this patch, the red stimulus with a green border, did not so strongly link with sour (50 such individuals), which relates to the topic of discussion next.

**Figure 4.**
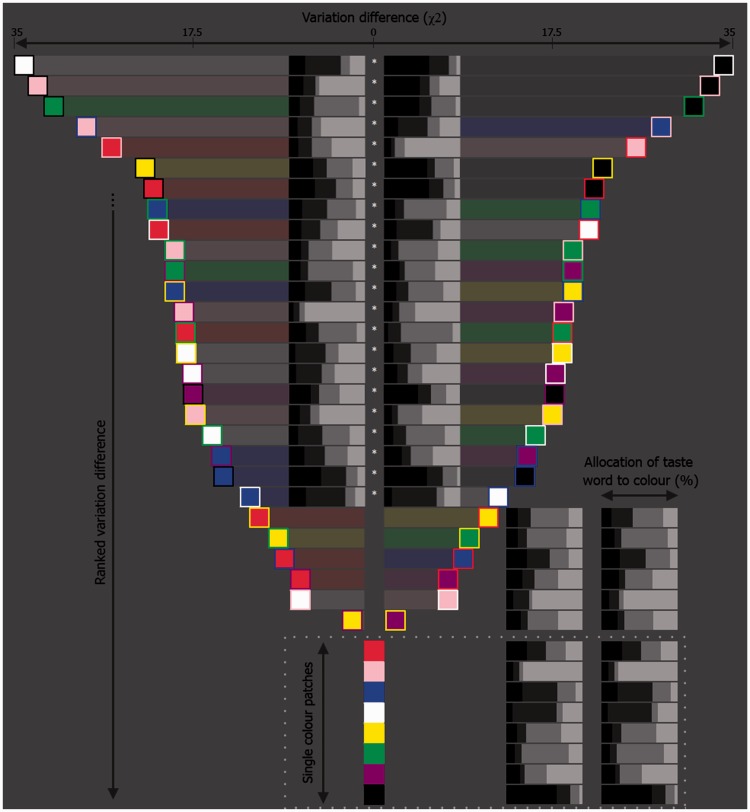
A color matrix plot depicting the extent to which color patches of X foreground color and Y border color differ from those of Y foreground and X border colors, differ in terms of patch to taste association differences (2 × 2 χ^2^ test value) to the four basic tastes. The horizontal distance between each XY pair reflects this difference. For example, at the top of the figure are the whiteBlack and blackWhite patches which are the most horizontally separated, and thus, most differing in terms of how participants assigned each patch to each of the basic tastes. Space permitting, the actual percentage tastes-word associations to each word have been plotted between each XY pair, by means of accumulative bar plots (the boxes of ascending lightness in each bar reflect, respectively, the tastes of bitter, salty, sour, sweet); the chart closest to a given patch reflects that patch’s % taste associations. XY pairs consisting of more than one color that statistically differ in terms of their degree of difference have a white star inserted directly between them. Note that the eight single color patches at the bottom of the figure have been added for completeness, and also to make available to the reader, how each was assigned to each of the individual colors.

As predicted, there was evidence for our hypothesis that patches consisting of the same two colors (e.g., blue and yellow), but, with one patch having a particular color as the foreground (blueYellow), while the other patch had that color as the border (yellowBlue) would not necessarily have the same pattern of taste associations. As shown in Figure 4, which shows the degree of difference between pairs of such patches, 39.3% of such patches statistically differed in terms of their pattern of association with the basic tastes. For example, the pattern of association strength for blueYellow for bitter, salty, sour, sweet (respective scores were 21, 36, 32, 12) differed significantly from that of the yellowBlue patch (16, 16, 46, 22), χ^2^(3) = 13.21, *p* = .0043, *p_crit_* = .0045, *w* = .26.

**Figure 5.**
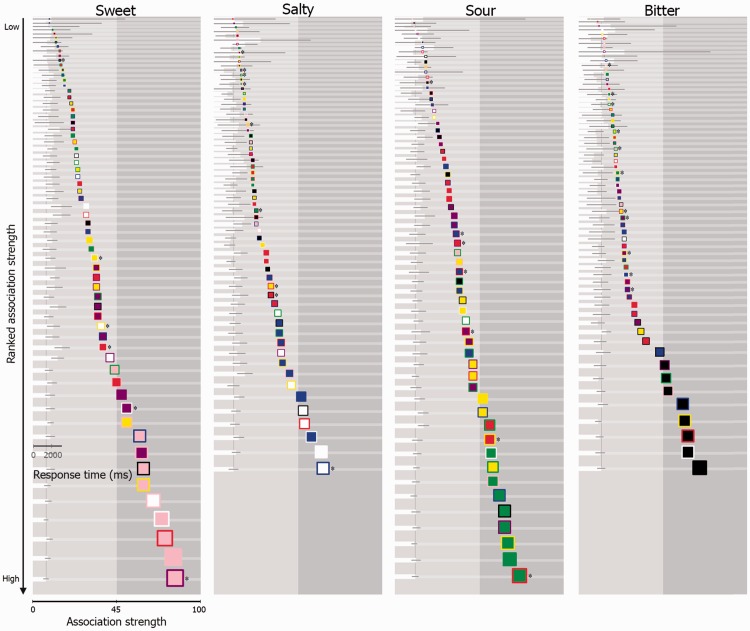
Four color matrix plots as specified in Figure 3. Now, the horizontal position of each patch represents the strength of the crossmodal association between the color or color pair, and a basic taste (as well as the size of each patch) with rightward-positioned patches being more strongly associated than those on the left-hand side (the more participants selecting a given color for a particular taste, the stronger the association; the horizontal axis being expressed as a ratio in terms of this value over the total number of participants). Single color patches were presented horizontally along the top and bottom of each plot. In terms of their vertical placement, double color patches are arranged from the weakest association (top) to the strongest (bottom) in each plot. For illustrative purposes, double color patches that were more strongly associated with a given taste than either of their constituent colors are suffixed with a star. The background of each plot was colored light and dark gray, the latter signifying the region in which a particular taste was selected for patches by participants at a level that was significantly greater than that expected by chance (chosen 45 or more times, *n* ≥ 45; *p* < .001; the experiment was simulated 10,000 times and the maximum number of consistent “by-chance” selections over all colors × tastes per virtual experiment was used to determine this). The average time taken by the participants to allocate a given color patch to a given taste has been plotted along the left-hand side of each of the four plots (bars represent the confidence interval around mean). The longer the time taken, presumably, the harder the participants found it to make their taste decision.

By means of a simulation (see Appendix A for the code; to run the simulation online visit http://try.haxe.org/#5F5d1), as shown in Figure 5, certain of the stimuli were selected more frequently for a given taste than expected by chance (stimuli falling in the darker gray box in each segment fit this criteria). As noted earlier, for bitter, the black stimulus with a black border is indeed shown to be the most strongly linked stimulus. Furthermore, as noted, the green stimulus with a red border most often links with sour. Indeed, this greenRed stimulus links more strongly with sour than either of its constituent colors (green and red)—a star has been suffixed to the right of each stimulus that has this property. Given the small degree of difference, however, we are not so sure that we would observe the same small differences if we replicated the study, given the sample size of Experiment 1.

**Figure 6.**
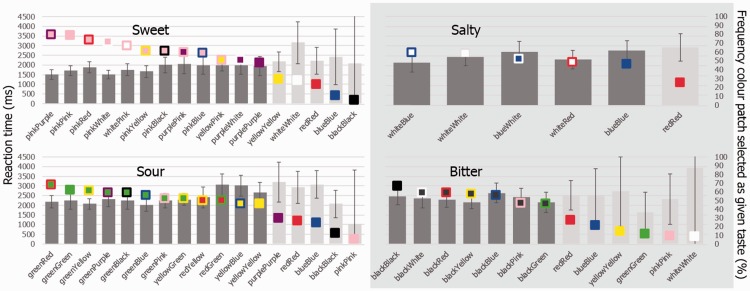
Time taken to assign a taste to a given color patch (represented via the bars) and frequency with which each patch was assigned as that taste (represented via the squares). Error bars represent the 95% CIs (note that the size of the error bars increases from left to right as the number of data points contributing to each bar decreases; this reflects the fact that the bars have been rank ordered according to frequency). Darker gray bars were those that were chosen at a level greater than that expected by chance (*n* ≥ 45; as defined in Figure 7). We have also included in the figure color patches that consisted only of a single color, when that given color was present in patches that *were* chosen by participants at a level shown greater than that expected by chance.

In terms of reaction times (RTs), by comparing confidence intervals, there were a range of two-color patches for each taste that did not take any longer to assign relative to the equivalent color patches of their constituent colors (see Figure 6). For example, referring to the green stimulus with a red border, it is worth noting that our participants did not take any longer to assign it as “sour” as compared with the purely green (or purely red) stimulus (the RT data are plotted in Figure 6). This might suggest that the green-red stimulus *better* “portrays” sourness than either of its constituents (and indeed, any other) colors. Note that for sweet, the pink with purple background stimulus, and for salty the white with blue background stimulus, are also both better able to portray the tastes they were most strongly associated with.

Thus, as hypothesized, some color patches were found to be more consistently linked with sweet (pinkPurple, purpleWhite), salty (whiteBlue), and sour (greenRed, redYellow), than their constituent colors, and at a level that was significantly greater than that expected by chance. As there was no difference in terms of RTs between color patches of a single color as compared with those double-colored (as the confidence intervals overlapped), it can be concluded that participants found it just as easy to link single colors with tastes as to link multicolor patches with tastes. Note that this was not the case in Woods and Spence’s (2016) study, where it took participants more than twice as long to assign a taste to side-by-side color pairs, than it did to single color patches.

We were curious to see whether the differences between the top five associated patches for each color were statistically discriminable with our sample size, by means of a power analysis (via G*Power, version 3.1.9.2, Faul, Erdfelder, Buchner, & Lang, 2009). One issue here was that the data for each taste were not independent, with, for example, a high number of assignments for a patch as sweet, salty, and sour leading to a low number of assignments as bitter. Although thus only approximate, and potentially influenced by how a patch was assigned to each of the other tastes, we decided to use the ratio of a given taste assignment for two patches (e.g., whiteBlue and whiteBlack were assigned as sweet 59 and 47 times, respectively, or .56 and .44; n.b. we chose these patches as out of all tastes, they differed most in terms of magnitude) to calculate that the required sample size was 1,015 data points (*w* = .11, α = .5, 1−β = .95; we currently have 106 data points assigned to these patches). About 10 times as many participants were found to be needed to reliably test for this effect. It stands to reason, then, that given the current sample size, there was no significant difference between whiteBlue and whiteBlack patches for saltiness, χ^2^(1) = 1.36, *p* = .24, *w* = .11.^2^

**Figure 7.**
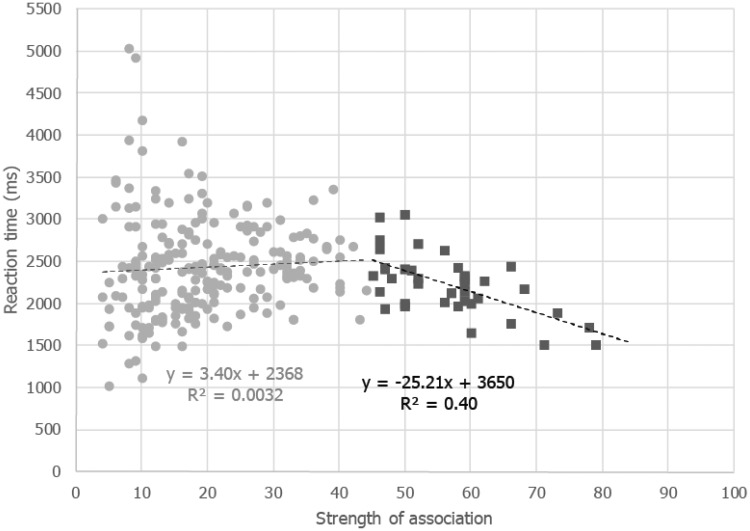
Relationship between the strength of association of the color patches with their respective tastes (*x*-axis) with the mean time taken for each patch to be assigned a taste (*y*-axis). The dark gray square dots are patches that were assigned to a given taste more often than expected by chance (see the legend of Figure 5 for this methodology).

The relationship between the time taken to assign each color to a taste and the number of times this assignment was chosen are plotted in Figure 7. Retrospectively, we split the data according to whether the number of assignments expected was above chance levels (≥45) or below this threshold (*p* < .001); it stands to reason that only when there were clear associations between color and tastes (i.e., above the threshold level), would one expect a relationship between RT and strength of association. Below the threshold, however, color-taste assignments would be more likely due to chance, and we would expect both RT and association strength to be rather random and unlikely correlate. When above the critical threshold, the dependent variables did indeed correlate significantly, *r_pb_* = −.57, *N* = 39, *p* = .0033, 95% CI [−.25, −.77]. Below the threshold, there was also a significant, albeit much weaker, relationship *r_pb_* = .14, *N* = 217, *p* = .0057, 95% CI [−.0043, .2722].

## Experiment 2

In Experiment 1, an order of magnitude more participants would have been needed to reliably distinguish between the top ranking color patches for each taste. The purpose of our second study was to see whether we could replicate the results of Experiment 1, but using a paradigm that we thought should be more sensitive to differences of association.

### Methods

#### Participants

One hundred participants born in the UK (48 women; *M*_age_ = 30.7 years, *SD*_age_ = 10.4, range_age_ = 18–60 years) were recruited from Prolific Academic to take part in the study in return for a payment of 0.50 UK pounds. The experiment was conducted on December 16, 2015 from 17:18 GMT onwards, over a period of 82 min, at a rate of 1.22 participant completions per minute. The participants took an average of 157 s (95% CI [142, 171]) to complete the study.

#### Stimuli and apparatus

The stimuli and apparatus were based on those used in Experiment 1. However, only the five most strongly associated color patches for salty (whiteBlue, whiteWhite, blueWhite, whiteRed, whiteBlack), bitter (blackBlack, blackRed, blackWhite, blackYellow, blackBlue), sweet (pinkPurple, pinkPink, pinkRed, pinkWhite, whitePink), and sour tastes (greenRed, greenGreen, greenYellow, greenPurple, greenBlack) from Experiment 1 were used as stimuli again here. As in Experiment 1, whether a given participant was shown circular or square stimuli was randomly determined.

#### Design

A within-participants experimental design was adopted with all of the participants undertaking all four of the experimental trials, in a random order. The dependent variable was the relative position, expressed as a percentage, between two anchor points that specified a given taste, for example, on the left as, “not sweet,” and on the right as “very sweet” (salty, bitter, and sour were the other tastes besides sweetness that were used).

#### Procedure

**Figure 8.**
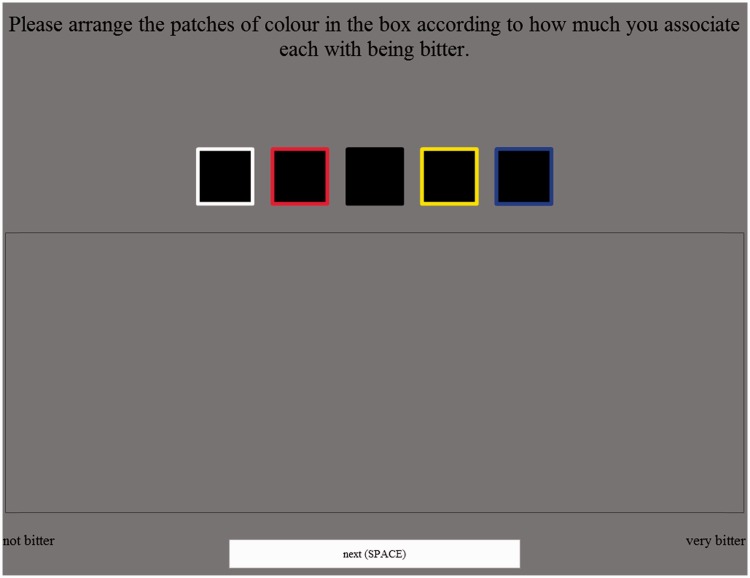
Screenshot illustrating the task used in Experiment 2 (this example shows the case of “bitter”).

A screen shot of the task is shown in Figure 8. The participants had to arrange five color patches within a box, placing each patch so that its horizontal position matched how intensely that they thought that each patch was in terms of a particular taste (this task has been used successfully in several recent studies, Velasco, Woods, Hyndman, & Spence, 2015). The original starting positions for the patches were above the box, with the colors arranged in a horizontal line with the patches being 102.4 pixels away from its neighbor(s). The order of the patches varied randomly across participants. After placing all five patches, the participant could then proceed on to the next trial by pressing the space bar or clicking the “next” button (there was a 100 ms pause between trials). On each of the four trials, a different taste was presented and a different set of patches were assessed. Trial order was random over participants. After completing all the trials, participants were debriefed as to the nature of the study.

### Results and Discussion

**Figure 9.**
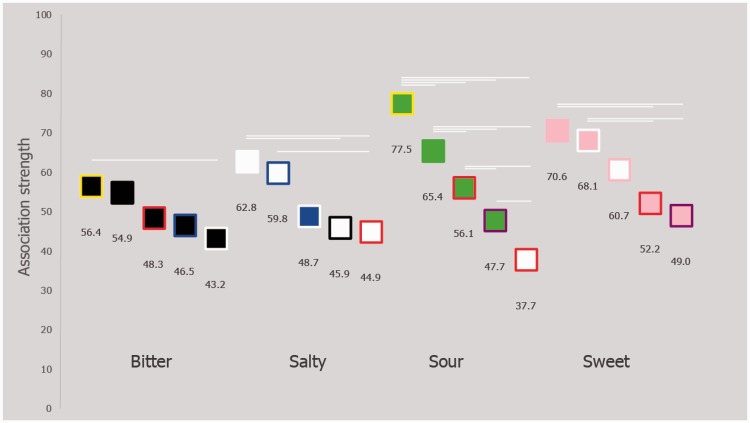
Illustrating the mean taste association strength rating for each color patch in Experiment 2. Horizontal white lines vertically linking two patches reflect that those two patches statistically differed in terms of taste association (*p* < .05; as detailed in Supplementary Table).

The effects of patch color on rated taste association strength (plotted in Figure 9) were tested for each basic taste seperately, in order to establish whether the mean differed amongst dependent groups of data. In contrast to the results of Experiment 1, there were statistically significant differences in terms of how each of the five sets of patches were rated for bitter *F*(3.62, 213.47) = 2.84, *p* = .030, salty *F*(2.83, 166.85) = 6.29, *p* < .001, sour *F*(3.58, 211.04) = 38.48, *p* < .001, and sweet *F*(2.74, 161.60) = 9.80, *p* < .001 (post hoc tests are reported in Supplementary Table). It would thus seem that this methodology was more sensitive to detecting subtle association differences between the patches, as compared with the methodology used in Experiment 1.

**Figure 10.**
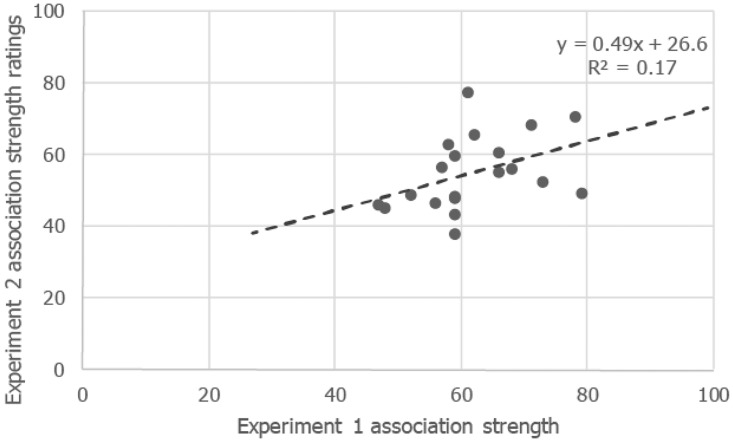
The relationship between association strength of the patches with tastes reported in Experiment 1, along with the mean taste association ratings for the different patches reported in Experiment 2.

As would be expected, the mean taste association strengths of the color patches in Experiment 2 correlated with their association strengths from Experiment 1, *r_pb_* = .48, *N* = 20, *p* = .0067, 95% CI [.13, .74] (the data are plotted in Figure 10). Given that the correlation could only account for 21.87% variation (or 17% via simple classic regression) other factors may have held sway over the scores. Of course, given that an order more participants would be required to reliably detect for differences between scores in Experiment 1, random variation could well explain this difference between studies.

One confound was identified, be it retrospectively: In Experiment 1, as participants could only assign one taste to each patch, if a given patch were to be strongly associated with two tastes, the consequence is that both tastes would receive many votes but fewer than they would if their were only one strongly associated taste. To test for this confound—henceforth termed the “Dilution confound”—it holds, in Experiment 1, that if a patch has a second “strongly associated” taste, then assignments to that second strongly associated taste do so at the expense of those of the most strongly associated taste. The sum of the most and second most strongly associated taste values would cancel out this effect and we would expect it to better correlate with the scores of Experiment 2. This was not the case, *r_pb_* = .43, *N* = 20, *p* = .053, 95% CI [−.02, .77]—the confidence intervals did not overlap. Do note though that the small sample sizes could well imply Type 1 error (where there is an underlying effect but there is insufficient data to robustly detect that effect). Another consideration is that a dilution effect should only be detectable when a patch is strongly linked to two colors, which has the knock on consequence of those two colors being seen (via the paradigm in Experiment 1) to only moderately associate with each color. None amongst our 20 top 5 *strongly* associating to taste color patches would thus likely be affected by a dilution effect.

There was no relationship between the time taken for participants to assign color patches to a given taste in Experiment 1, and the taste association reported in Experiment 2, *r_pb_* = −.20, *N* = 20, *p* = .39, 95% CI [−.59, .27].

## General Discussion

Taken together, the results of the two experiments reported in the present study provide an interesting contrast with those reported recently by Woods and Spence (2016). In the latter study, where pairs of equally sized color squares were placed side-by-side, the participants may have struggled to determine which color should determine their response, presumably because both colors were given an equal weighting in the decision process. In the present study, by contrast, it was more obvious that the foreground color was the primary color to match to the named taste (perhaps because foreground elements are processed more rapidly than those in the background, Lester et al., 2009). Hence, when both the foreground and background colors were associated with the same taste, participants responded more confidently (i.e., more rapidly) than when presented with the best of the individual colors. We could also conclude though that the color combinations used here were perceived as just one combined element, as opposed to separate conflicting elements (in Woods & Spence, 2016).

As before, some color combinations were once again found to be easier for people to associate with certain basic tastes (e.g., sweet, bitter, salty, sour). Additionally, these combinations *did not* take any longer to match with a given taste, thus indicating that they would perhaps be better suited to conveying taste properties as compared with the unique colors when presented in isolation. An interesting point for future consideration is whether this finding would change if the ratio of border thickness (currently 5 pixels) relative to overall patch width or height (50 pixels) were to be varied (cf. Ou, 2015).

It was encouraging that the data from the different paradigms used Experiments 1 and 2 correlated with each other. This relationship could only account for 21.87% variation however, implying other factors could be at play. We tested whether Experiment 1 was affected by a dilution confound but found no real evidence for this; we argued though that we made it very unlikely to detect such affects by restricting ourselves to the top five patches from Experiment 1. Indeed, as raised by a keen-eyed reviewer, another consequence of us using the top five most strongly associated patches from Experiment 1, and not patches of a range of association strengths, is that we restricted the *range* of our stimuli, which very likely reduced the strength of any association between the experiments (e.g., there may be no relationship between intelligence quotient scores and mathematical ability for members of the high intelligence quotient society, Mensa, but such a relationship may exist in the general population; see, Kantowitz, Elmes, & Roediger, 2008, p. 42). In a similar vein, another potential explanation is based on the fact that crossmodal correspondences are sensitive to stimulus context that may arise from the relative compatibility of the bimodal component stimuli (Martino & Marks, 2001; Parise, 2016; Parise & Spence, 2013). In other words, one color may be more likely to be associated with a taste, relative to the other colors and tastes in the experiment. It is also worth considering whether seeing all the pairs of colors together in a single trial could have swayed the results. For example, if one combination “stood out from the crowd” this could well have confounded results (e.g., for sourness, all patches had a green interior except for one whose interior was white—could this distinction have contributed to this particular patch being rated as the patch *least* associated with sourness?). Of course, given that we found Experiment 1 would need an order more participants to reliably distinguish between high scoring patches, random variation probably played a large role here as well.

In both of the experiments reported here, pink, white, green, and black foreground colors were associated with sweet, salty, sour, and bitter, which is consistent with the previous color-taste matches reported in the review on the topic by Spence et al. (2015). Border color, on the other hand, acted to modulate the strength of the association between the patch and taste (see Figures 5 and 9). For example, in Experiment 1, the top five matches to the word “sweet” were pink bordered with purple, red, pink, and white, and white bordered with pink (Figure 5), and, by means of Experiment 2, some of these patches did indeed differ in terms of strength of association (Figure 9). Needless to say, there are likely a whole range of additional factors that can sway such associations, a good first step to take in future research would be to focus on manipulating the saliency of the component colors of the patches, or even combining more than two colors. Tangentially, and demonstrating this tailoring effect of concept of complex color combinations, Jacquot, Noel, Velasco, and Spence (2016) have found that Lorraine University’s Chromatic Cards (patent FR n1255688)—shapes composed of different complex color arrangements in single patches—reliably match with specific odours (see also Schifferstein & Howell, 2015).

One take-home message here then is that if one’s goal is to identify the best variant amongst many, in terms of linking to a given concept, one could use the paradigm in Experiment 1 but collect a much larger number of participants (e.g., 1,000 in total). Or, as done here, one could get a preliminary sense of which patches are best linked with a concept by means of the paradigm outlined in Experiment 1, and then conduct a focused follow-up study on the high scorers, by means of the paradigm developed in Experiment 2 (which required 200 participants over both studies). This could be potentially interesting to those marketers and product designers wanting to combine color cues to convey information about a given product feature. With the available platforms for online research (Woods et al., 2015), it is now easy to rapidly collect data to test a given design decision; you see, although most of the time the designer’s intuition will be spot on, on occasion, for whatever reason, the public’s impression will differ from that of the designer, which, for example, could lead to a new product failing on launch. Corroborating this view, in the related discipline of high cuisine, we recently demonstrated that the culinary “law” of an odd number of food items on the plate is best, does not seem to be correct (Woods, Michel, & Spence, 2016).

The experimental technique outlined here (and in our previous article; Woods & Spence, 2016), of testing large numbers of participants online, to hopefully find the ideal permutations from amongst many possible alternative colors and color combinations, can be readily extended to other scenarios. For instance, one could imagine that instead of finding ideal color combinations to match each of the basic tastes, ideal combinations could also be found, say, for the colors or shapes that should be used in a logo (i.e., for a sweet-tasting new fruit juice). The technique could readily be applied to those situations where one needs to search for optimal solutions over many possible permutations. However, although this approach could potentially be extended to search amongst stimuli with three or even four varying features (see Favre & November, 1979), when more features need to be covaried, or varied continously rather than by discrete (few) steps, the number of permutations rapidly becomes too many to individually test. Potentially, employing guided evolutionary algorithms may be one means of exploring such a feature scape (Eiben & Smith, 2015). We turn our attention to this methodology next.

## Supplementary Material

Supplementary material

## Notes

1 A consideration is that two clashing colors (be it perhaps in terms of harmony, see Ou, 2015, for a review) may actually be more closely associated with a given taste, not directly by means of the color-taste associations, but rather by emotional valence (cf. Marks, 1996; Schloss & Palmer, 2011).

2 We conducted further analyses to test whether, as hypothesized, the rectangular color patches would be less strongly associated with sweetness (and more strongly associated with the other tastes), while the rounded patches would be less strongly associated with the other tastes (but more strongly associated with sweet). In particular, we ran eight separate Holm-Bonferroni corrected 2 × 2 chi-square tests, one for each interior patch color (we could not include the border color as an additional factor in an analysis as this would introduce many “empty cells”). The aim of this test was to investigate whether taste assignment (salty, sweet, bitter, and sour) varied between rounded and rectangular shapes. Only pink χ^2^(3) = 11.14, *p* = .011, did so at a level below that predicted by chance. Unexpectedly, square patches (12%, 12%, 11%, 65%, respectively linked with bitter, salty, sour, sweet) were more consistently linked with sweetness than circular patches (13%, 13%, 13%, 61%). Although interesting, the magnitude of difference is very small (perhaps reflecting a Type 1 error). We therefore decided not to speculate on this further here; we are though planning to explore this in the future via a dedicated experiment (as it contrasts with previous findings, see Velasco et al., 2015).

## Appendix A

The Haxe (version 3.2.1) code used to conduct the simulation in Experiment 1. You can run the simulation in your browser http://try.haxe.org/#5F5d1

class Test **{**

static **function** main**() {**

Sim**.**DO**();**

**}**

**}**

class Sim**{**

public static **var** SJs**:**Int** = **102**;**

public static **var** sims**:**Int** = **1000**;**

public static **var** singleColours**:**Array**<**String**> =**

"pink,purple,blue,red,white,yellow,green,black"**.**split**(“**,”**);**

public static **var** colours**:**Int** = **64**;**

public static **var** tastes**:**Array**<**String**> = **"salty,bitter,sour,sweet"**.**split**(“**,”**);**

public static **function** DO**(){**

**var** likelihood**:**Array**<**Int**> = new** Array**<**Int**>();**

**for(**a **in** 0 … SJs**){**

likelihood**.**push**(**0**);**

**}**

**for(**sim **in** 0 … sims**){**

likelihood**[**OneExpt**.**DO**()]++;**

**}**

trace**(‘**count p'**);**

**for(**count **in** 0 … likelihood**.**length**){**

trace**(**count **+**' ‘**+**likelihood**[**count**]/**SJs**);**

**}**

**}**

**}**

class OneExpt**{**

public static **function** DO**():**Int**{**

**var** result**:**Stimulus**;**

**var** highestFreq**:**Int** = **0**;**

**for(**taste **in** 0 … Sim.tastes.length**){**

**for(**colour1 **in** 0 … Sim.singleColours.length**){**

**for(**colour2 **in** 0 … Sim.singleColours.length**){**

result** = **getFrequencies**();**

**if(**result**.**highest** > **highestFreq**)** highestFreq** = **result**.**highest**;**

**}**

**}**

**}**

**return** highestFreq**;**

**}**

private static **function** getFrequencies**():**Stimulus**{**

**var** stim**:**Stimulus** = new** Stimulus**();**

**for(**sj **in** 0 … Sim.SJs**){**

stim**.**freq**[**OneTrial**.**DO**()]++;**

**}**

stim**.**calc_highest**();**

**return** stim**;**

**}**

**}**

class OneTrial**{**

public static **function** DO**():**Int**{**

**return** Std**.**int**(**Math**.**random**() *** Sim**.**tastes**.**length**);**

**}**

**}**

class Stimulus**{**

public **var** colour1**:**String**;**

public **var** colour2**:**String**;**

public **var** taste**:**String**;**

public **var** freq**:**Array**<**Int**> = new** Array**<**Int**>();**

public **var** highest**:**Int**;**

public **function new(){**

**for(**taste **in** 0 … Sim.tastes.length**){**

freq**.**push**(**0**);**

**}**

**}**

public **function** calc_highest**(){**

**var** high**:**Int** = **0**;**

**for(**i **in** freq**){**

**if(**i** > **high**)**high** = **i**;**

**}**

highest** = **high**;**

**}**

**}**
