# Supplementary material for: Using Single Colors and Color Pairs to Communicate Basic Tastes II: Foreground–Background Color Combinations
Source: Iperception. 2016 Sep 20;7(5):2041669516663750. doi: 10.1177/2041669516663750 (PMC5034334; doi:10.1177/2041669516663750)
Supplement: Supplementary material [file Supplementary_Table.pdf]

Supplementary Table: Showing the results of the posthoc tests from Experiment 2, conducted with the rmmcp function as part of the 'WRS' r package. The test compares the mean among dependent variables and ascertains whether they differ more so than expected by chance. The test controls for multiple comparisons (hence the 'critical *p*' column in the table).

Taste	Patch 1	Patch 2	T	*p*	Critical *p*	*SE*	Sig diff
bitter	blackBlack	blackRed	1.38	0.17193	0.0127	4.21	
	blackBlack	blackWhite	2.6	0.01166	0.00568	3.41	
	blackBlack	blackYellow	-0.35	0.72541	0.05	5.21	
	blackBlack	blackBlue	2.46	0.01689	0.00639	3.53	
	blackRed	blackWhite	1.52	0.13499	0.00851	3.89	
	blackRed	blackYellow	-1.68	0.09908	0.0073	3.53	
	blackRed	blackBlue	0.39	0.69715	0.025	2.95	
	blackWhite	blackYellow	-3.52	0.00085	0.00511	3.58	*
	blackWhite	blackBlue	-0.73	0.4702	0.0169	3.79	
	blackYellow	blackBlue	1.47	0.14741	0.0102	4.5	
salty	whiteBlue	whiteWhite	-1.45	0.15146	0.0127	4.07	
	whiteBlue	blueWhite	2.59	0.01217	0.00851	3.18	
	whiteBlue	whiteRed	4.11	0.00013	0.00511	3.17	*
	whiteBlue	whiteBlack	2.68	0.00952	0.0073	4.09	
	whiteWhite	blueWhite	2.3	0.02506	0.0102	6.16	
	whiteWhite	whiteRed	3.77	0.00038	0.00568	4.52	*
	whiteWhite	whiteBlack	3.41	0.00119	0.00639	3.95	*
	blueWhite	whiteRed	0.14	0.89111	0.05	4.95	
	blueWhite	whiteBlack	0.24	0.80814	0.025	5.62	
	whiteRed	whiteBlack	-0.41	0.68646	0.0169	3.74	
sour	greenRed	greenGreen	-2.35	0.02211	0.025	4.27	*
	greenRed	greenYellow	-5.98	0	0.0073	3.43	*
	greenRed	greenPurple	2.14	0.03652	0.05	3.49	*
	greenRed	greenBlack	3.69	0.00049	0.0127	3.85	*
	greenGreen	greenYellow	-3.66	0.00055	0.0169	2.75	*
	greenGreen	greenPurple	4.95	0.00001	0.00851	3.79	*
	greenGreen	greenBlack	6.31	0	0.00639	4.06	*
	greenYellow	greenPurple	7.12	0	0.00568	3.82	*
	greenYellow	greenBlack	10.38	0	0.00511	3.71	*
	greenPurple	greenBlack	3.8	0.00034	0.0102	2.87	*
sweet	pinkPurple	pinkPink	-5.75	0	0.00511	3.67	*
	pinkPurple	pinkRed	-0.85	0.39956	0.025	2.76	
	pinkPurple	pinkWhite	-4.41	0.00004	0.00568	4.32	*
	pinkPurple	whitePink	-2.6	0.01179	0.00851	4.76	
	pinkPink	pinkRed	3.54	0.00078	0.00639	5.18	*
	pinkPink	pinkWhite	0.7	0.48425	0.05	2.99	
	pinkPink	whitePink	2	0.05039	0.0102	4.03	
	pinkRed	pinkWhite	-3.38	0.00128	0.0073	4.69	*
	pinkRed	whitePink	-1.65	0.10504	0.0169	5.85	
	pinkWhite	whitePink	1.79	0.07873	0.0127	2.86	
